# Pathogenic roles of follicular helper T cells in IgG4-related disease and implications for potential therapy

**DOI:** 10.3389/fimmu.2024.1413860

**Published:** 2024-06-07

**Authors:** Jingyi Xu, Jiayu Zhai, Jinxia Zhao

**Affiliations:** ^1^ Department of Rheumatology and Immunology, Peking University Third Hospital, Beijing, China; ^2^ Center for Rare Disease, Peking University Third Hospital, Beijing, China

**Keywords:** follicular helper T cells, T lymphocyte, IgG4-related disease, pathogenesis, therapeutic targets

## Abstract

IgG4-related disease (IgG4-RD) is a recently described autoimmune disorder characterized by elevated serum IgG4 levels and tissue infiltration of IgG4^+^ plasma cells in multiple organ systems. Recent advancements have significantly enhanced our understanding of the pathological mechanism underlying this immune-mediated disease. T cell immunity plays a crucial role in the pathogenesis of IgG4-RD, and follicular helper T cells (Tfh) are particularly important in germinal center (GC) formation, plasmablast differentiation, and IgG4 class-switching. Apart from serum IgG4 concentrations, the expansion of circulating Tfh2 cells and plasmablasts may also serve as novel biomarkers for disease diagnosis and activity monitoring in IgG4-RD. Further exploration into the pathogenic roles of Tfh in IgG4-RD could potentially lead to identifying new therapeutic targets that offer more effective alternatives for treating this condition. In this review, we will focus on the current knowledge regarding the pathogenic roles Tfh cells play in IgG4-RD and outline potential therapeutic targets for future clinical intervention.

## 1 Introduction

IgG4-related disease (IgG4-RD) is a systemic fibro-inflammatory disease affecting multiple organs, characterized by a significant elevation in serum IgG4 concentration and infiltration of IgG4-positive plasma cells into affected tissues (1–3). The histopathological features of IgG4-RD lesions include lymphoplasmacytic infiltration, obliterative phlebitis, and storiform fibrosis (1). Within two decades, since IgG4-RD was originally recognized as a distinct systemic multi-organ disease entity in patients with autoimmune pancreatitis, substantial progress has been achieved in the field of IgG4-related disease (4, 5). Recent advancements in the pathophysiology have shed light on the immunological mechanism in IgG4-RD, highlighting the dysregulation of various T and B cell subsets along with their cytokines as contributors to tissue damage and fibrosis (6). Despite extensive research in identifying the underlying immunopathogenesis, the etiology of the disease remains incompletely understood. Currently, available conventional therapies such as glucocorticoids and immunosuppressors have notable limitations. Although significant strides have been made in identifying treatments for this condition over recent decades, it still lacks curability with a high risk of recurrence. Therefore, further efforts are imperative to improve the prognosis of IgG4-RD. Consequently, enhancing our comprehension of the pathogenesis is of paramount importance for developing more potent and specific treatments while identifying novel therapeutic targets. Emerging evidence suggests that follicular helper T (Tfh) cells may play a critical role in the pathogenesis of IgG4-RD. Moreover, recent insights into the pathological roles played by Tfh cells offer promising potential for targeting these cells to optimize therapeutic strategies.

Tfh cells, a subset of CD4^+^ T cells, play a crucial role in GC formation, B cell expansion, and differentiation for antibody production (7, 8). They are characterized by their distinct features of high expression of surface molecules, including the chemokine receptor CXCR5, the costimulatory molecule ICOS, and the coinhibitory molecule PD-1 (9). Tfh cells are regulated by multiple transcription factors, including B cell lymphoma 6 (BCL-6), and exhibit a distinct cytokine production pattern, notably IL-4 and IL-21 (10). They are predominantly localized in lymphoid organs, although they can also be found in peripheral blood and lesions of diseases (11). Based on their distribution, Tfh cells can be classified into two groups: Tfh cells in GCs and circulating follicular helper T (cTfh) cells in peripheral blood. More specifically, according to the expression patterns of chemokine receptors, the cTfh population can be further divided into three major subsets: Tfh1 (CXCR3^+^ CCR6^-^), Tfh2 (CXCR3^-^ CCR6^-^), and Tfh17 (CXCR3^-^ CCR6^+^) (12, 13). Functionally, Tfh cells contribute to antibody affinity maturation, IgG4 class-switching, and somatic hypermutation by assisting B cell development and plasma cell differentiation (14). The substantial accumulation of Tfh cells at affected sites provides compelling evidence of their active role in the pathogenesis of the disease (15). Excessive expansion and dysfunctional activity of Tfh cells have been associated with aberrant immune response and disease progression, leading to tissue damage and IgG4 production in individuals with IgG4-RD (7).

**Figure 1:**
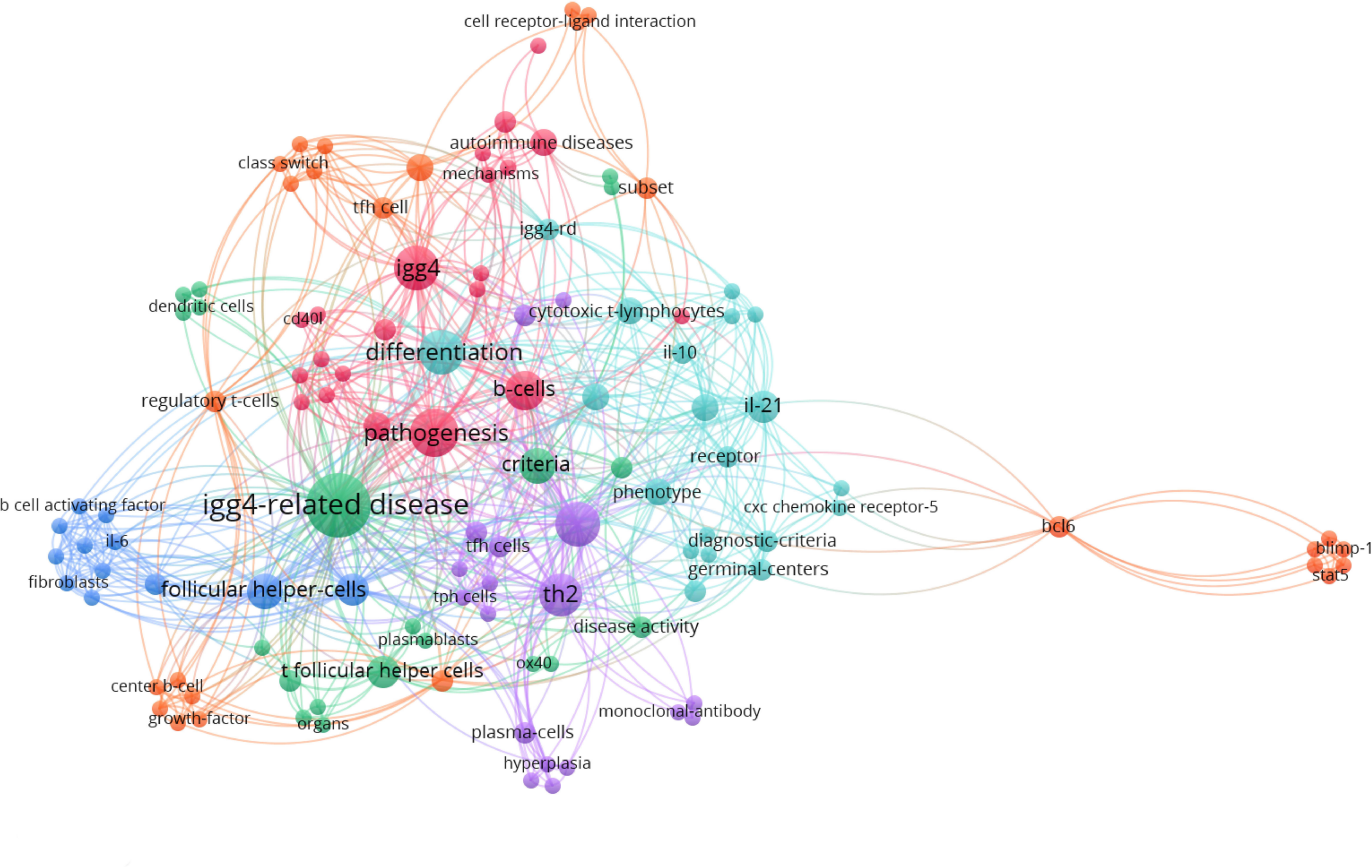
Clusters of keywords co-occurrence analysis of the most recent publications regarding Tfh and IgG4-related disease with VOS viewer.

The co-occurrence analysis of keywords using VOS viewer software has effectively summarized the latest advancements related to Tfh in IgG4-RD, depicting the participation of T cell subsets in the pathogenesis of IgG4-RD as well as suggesting potential molecule targets for targeted therapeutic interventions. (**Figure 1**) This review aims to elucidate the pathogenic roles of Tfh cells in IgG4-RD by focusing on the recent progress, thereby providing valuable insights into the function of Tfh cells in IgG4-RD pathogenesis and identifying potential therapeutic targets associated with Tfh cells.

## 2 Pathophysiology

### 2.1 Molecules of Tfh cells associated with the pathogenesis of IgG4-RD

Tfh cells undergo several stages of differentiation when they migrate to the T-B border and interact directly with antigen-specific B cells (16). Throughout this process, the expression of multiple molecules is upregulated for intercellular recognition and signal transduction, which play an essential role in Tfh-B interaction as well as the pathogenesis of IgG4-RD (17). The increased expression of Tfh-related molecules in affected tissues is essential for the pathogenesis of IgG4-RD (18, 19). An in-depth exploration into the molecular mechanisms of Tfh differentiation may provide novel perspectives to the treatment for IgG4-RD.

#### 2.1.1 CXCR5

CXCR5 serves as a surface marker of Tfh cells, facilitating their migration into germinal centers through interaction with its ligand CXCL13. CXCL13 is produced by follicular stromal cells at high levels within B-cell follicles (20). The crucial interactions between CXCR5 and its ligand CXCL13 are indispensable for promoting Tfh cell migration and their interaction with B cells in GCs (21). Enhanced expression of both CXCL13 and CXCR5 in affected tissues of IgG4-RD indicated their participation in Tfh migration and ectopic germinal center formation (22). Noticeably, Tfh cells can also produce CXCL13, therefore contributing to germinal center formation in inflamed tissue. Given the significant role of CXCL13-CXCR5 chemokine axis in follicular reaction, insights from one study considered CXCL13 an important plasma biomarker of germinal center activity (23).

#### 2.1.2 ICOS

ICOS/ICOSL is another signaling pathway that maintains Tfh cell differentiation and facilitates GCs formation, T-B interactions, and antibody production (24), while PD-1/PD-L1 signaling acts as a counterbalance by inhibiting ICOS signaling and restraining Tfh cell proliferation, thereby preventing excessive B cells proliferation and antibody production (20). In IgG4-RD, it has been observed that infiltrating submandibular glands express ICOS on Tfh cells, which enhances their ability to assist B cells in producing IgG4 (25). Furthermore, engagement between ICOS and its ligand on B cells can in turn trigger and sustain the differentiation of Tfh cells.

#### 2.1.3 PD-1

PD-1 plays a crucial role in the selection and maturation of B cells within GCs, making it a potential marker for cell activation. Dysfunction of PD-1 may be associated with the development of IgG4-RD (26). A study has demonstrated that PD-1 expression in cTfh cells positively correlates with serum IgG and IgG4 levels, IgG4:IgG ratio, number of involved organs, and frequency of plasmablasts (27). Recent findings have highlighted the significance of PD-1^+^Tfh2 cells in providing B-cell assistance through direct interaction and cytokine production in IgG4-RD, suggesting that PD-1 holds promise as both a disease biomarker and a potential target for immunotherapy (25).

#### 2.1.4 CD40L

In germinal center Tfh-B interactions, the interaction between CD40L expressed by Tfh cells and CD40 on B cells plays a pivotal role in providing signals for B cells proliferation, differentiation, and antibody class switching, ultimately contributing to GC formation (28, 29). In one case report, expression of CD40 and its ligand CD40L was detected to be augmented in affected lesions of IgG4-RD patients, which indicated the activation of the signaling pathway involved in Tfh-B interactions (18).

#### 2.1.5 OX40

The activation of OX40 by OX40L serves as a T-cell co-stimulator, while OX40/OX40L signaling plays a role in Tfh cell differentiation and improves the helper function of Tfh for B cells (30). Recent research has focused on exploring the potential therapeutic benefits of both agonists and blockers targeting the OX40-OX40L interaction for treating T cell-mediated autoimmune diseases, which have attracted much attention (31, 32).

#### 2.1.6 Transcription factors

The activation and differentiation of Tfh are also crucial at transcriptional levels, which involves the activation and regulation of multiple transcription factors, including positive factors such as Bcl-6 and Batf, as well as negative factors such as Blimp-1. Bcl-6 and Blimp-1 play critical roles in regulating Tfh development and differentiation but exhibit antagonistic effects (33). Bcl-6 is highly expressed in ectopic GCs of affected tissues in IgG4-RD patients, especially sinus tissues and gland tissues (27, 34), which initiates the early-stage differentiation of Tfh cells (35). Conversely, upregulation of Blimp-1 is mainly observed in Tfh cells in the peripheral blood (27), inhibiting both Bcl-6 expression and Tfh cell differentiation (36). Batf is also essential in the differentiation of Tfh cells and is connected to switched antibody responses (37). Batf^+^Tfh cells are abundant in tissues of patients with IgG4-RD (38), playing a positive role in promoting IL-4 production (39).

### 2.2 Tfh and B cells in affected tissue and ectopic GC formation

The presence of numerous ectopic germinal centers (GC) in affected tissues is considered to be a typical pathological characteristic of IgG4-RD (25, 40, 41). Histologically, a GC typically consists of two regions, the dark zone, where B cells undergo proliferation, and the light zone, where follicular dendritic cells (FDCs) and CD4^+^Tfh cells are located (42). FDCs can produce CXCL13 and contribute to ectopic GC formation by interacting with CXCR5, therefore leading to the accumulation of Tfh cells and B cells in affected sites of IgG4-RD (43). In GCs of lymphoid organs, Tfh cell assistance is essential to provide signals necessary for B cell proliferation and affinity selection. By enhancing IL-4 production and expression of surface molecules such as CD40L and ICOS, Tfh cells assist the expansion of naive B cells and their differentiation into high-affinity memory B cells and long-lived plasma cells (7). Additionally, Tfh cells secrete IL-21 and induce the transcription factor Bcl-6 expression to facilitate neighboring GC-B cells in completing class-switch recombination and affinity maturation (44). Abnormal expansion or dysfunction of Tfh cells may lead to ectopic GC formation and excessive immune responses associated with IgG4-related disease (8).

A study demonstrated that Tfh cells constituted over 70% of CD4^+^ T cells infiltrating the salivary gland in IgG4-RD (15). Specifically, in one case of IgG4-RD, Tfh cells were observed infiltrating the GC light zone (45). In an analysis of tissue samples from patients with IgG4-RD, Tfh cells were predominantly located around glandular cells or within ectopic GCs and diffusely infiltrated the affected sinus tissue. Comparably, few Tfh cells were detected in tissues of non-IgG4-RD patients (27). Furthermore, the upregulated expression of Tfh-related molecules, such as CXCR5 and Bcl-6, was also identified in ectopic GCs from patients with IgG4-RD. Research has suggested that excessive production of Tfh cytokine IL-21 was associated with the formation of ectopic GCs in IgG4-RD, correlating with their abundance as well as IgG4/IgG ratio (34).

Much attention lately has also been drawn to the pathogenic subsets of B cells infiltrating and accumulating in affected sites of IgG4-RD. Recent studies have revealed the expansion of activated naïve B cells and IgD^-^CD27^-^ double-negative (DN) B cells in IgG4-RD using immunofluorescence analyses (46). They are considered as precursors of activated antibody-secreting cells (47, 48). DN B cells with high expression of CD80 and CD86 were indicated to interact with infiltrating T cells. Specifically, DN3 B cells are reported to be abundant and clonally expanded in IgG4-RD lesions, contributing to tissue inflammation and fibrosis (49). Furthermore, it is also suggested that extrafollicularly derived DN B cells with low expression of CXCR5 are correlated with production of pathogenic autoantibodies and IgG4 isotype switch by linking to pre-GC Tfh cells (46). Further elucidation of T and B cell subsets within affected sites will enable more precise targeting therapies for clinical intervention.

### 2.3 Participation of Tfh in plasmablast differentiation and IgG4 production

During follicular response, Tfh cells play a pivotal role in driving the differentiation of naïve B cells into plasmablasts before causing IgG4-related immune responses (50). Circulating plasmablast levels have been remarkably increased in patients with active IgG4-RD, showing a correlation with disease activity (40, 51, 52) and the extent of organ involvement (53). Given that the proportion of plasmablasts correlate with that of Tfh cells (54), this connection between plasmablasts and Tfh cells can be described as the plasmablast-Tfh cell axis (55). Meanwhile, the proportions of plasmablasts and Tfh cells have both experienced a significant decrease under glucocorticoid treatment in IgG4-RD (56, 57). A recent study even identified the baseline count of plasmablasts as an independent predictive factor of disease relapse among patients treated with glucocorticoids (58). Therefore, besides serving as a valuable biomarker for disease activity assessment, plasmablast counts also provide insights into treatment response and disease relapse. Controlling the Tfh cell-plasmablast axis may offer a potential therapeutic strategy for managing IgG4-RD.

Elevated levels of IgG4 are considered a characteristic feature of IgG4-RD, although the precise underlying mechanism remains incompletely understood. One study illustrated that the expanded cTfh cells in IgG4-RD are associated with the proliferation of IgG4-producing B cells and class switch to IgG4 (25). Another report also found positive correlations between the number of activated cTfh cells and serum IgG4 levels (59). In addition, according to one recent study, the number of IL-4^+^ Tfh cells in affected tissues of IgG4-RD patients positively correlated with serum IgG4 concentrations and IgG4/IgG-positive cell ratios (60). To be more specific, analysis of Tfh subsets has demonstrated that the subset of cTfh2 cells have a closer relation to IgG4 production and class switch (27). Consistent with these findings, another research also reported an increased number of cTfh2 correlating with serum IgG4 levels and IgG4/IgG ratio (55). Therefore, it can be concluded that Tfh cells, especially the Tfh2 subset, play an important role in IgG4 production and class-switch recombination.

### 2.4 Tfh-related cytokines contributing to IgG4 class switch recombination

Generally, class switch recombination involves rearrangement of B cell DNA that leads to a change in antibody class while remaining specificity. In class switch towards IgG4, cytokines of Th2, such as IL-4 and IL-13, and regulatory T (Treg) cytokines, including IL-10, are suggested to be associated with the production of IgG4 antibodies and antibody response towards IgG4 (50). In patients with IgG4-RD, this process also involves the participation and activation of multiple cytokines produced by disease-specific Tfh cells, such as IL-4, IL-10, and IL-21 (61). Tfh2 cells can produce IL-4 and IL-21, therefore are presumed to play a role in IgG4 class switch (38, 62, 63). According to results of one experiment, IgM-positive B cells can be stimulated by T cells and switch to IgG4 and IgE with the presence of IL-4 (12, 64). Research has proved an elevation of both IgG4 levels and IgG4/IgG ratio under IL-4 stimulation, confirming that IL-4-induced IgG4 class switch may be one of the mechanisms in the pathogenesis of IgG4-RD (38, 63). Furthermore, IL-10 and IL-21 expressing Tfh cells were reported to be abundant in affected lesions from IgG4-RD patients. Evidence from experiments *in vitro* indicated that IL-10 can facilitate class switch mediated by IL-4 into IgG4 instead of IgE, therefore contributing to IgG4 class switch (61). In addition, IL-21 is another important cytokine secreted by Tfh cells, which regulates B cell expansion and Tfh cell development in GC formation and IgG4 class switch (65). Numerous studies have mentioned its pivotal role in IgG production in IgG4-RD (34, 66, 67).

**Figure 2:**
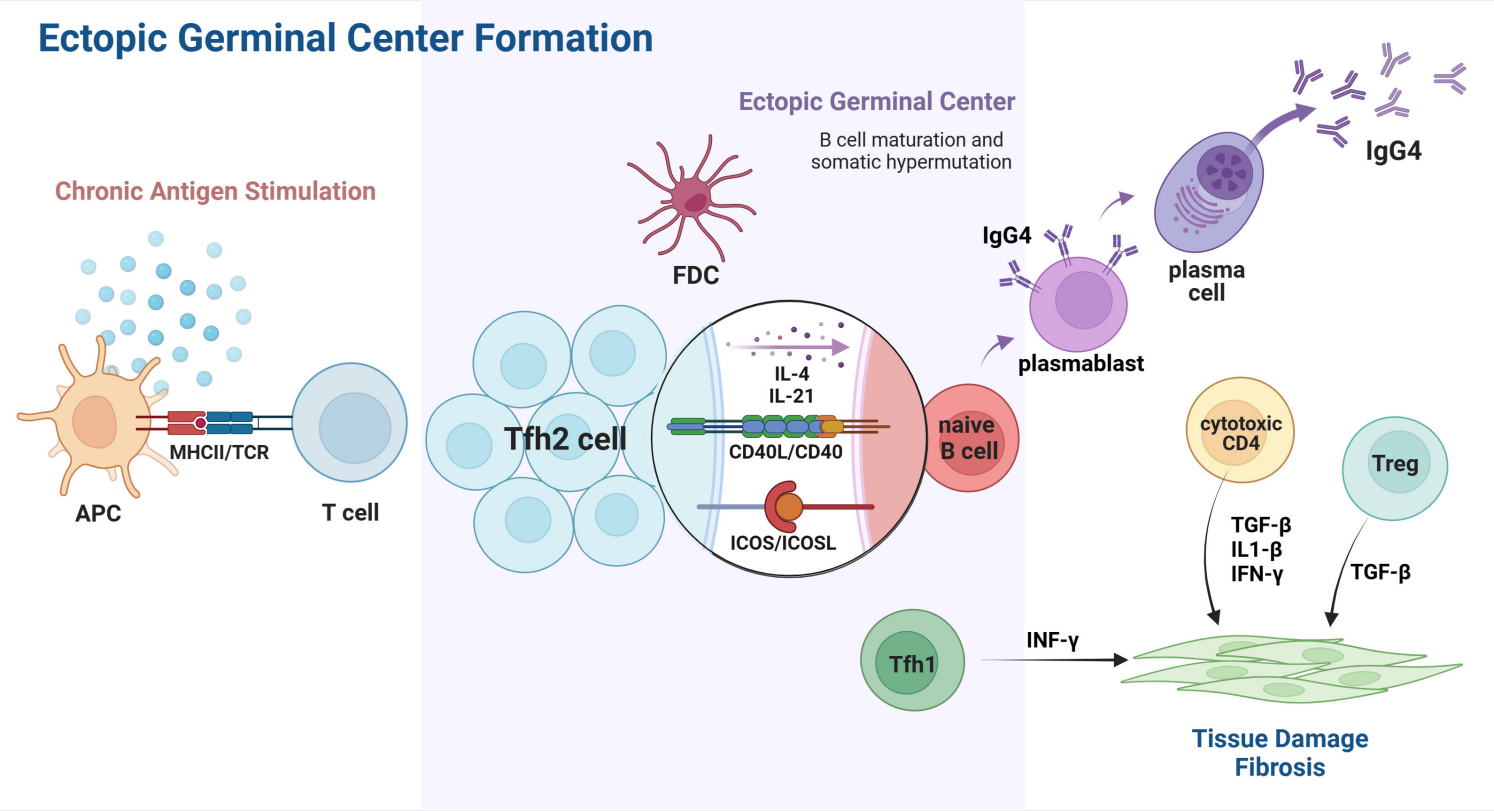
Tfh cells involved in ectopic GC formation of IgG4-RD. Chronic exposure to unknown antigens activates APCs and leads to T cell differentiation and polarization. The interaction of CXCR5 and CXCL13 promotes Tfh2 migration into the B-cell-rich zone, which eventually leads to the accumulation of Tfh2 and B cells, contributing to GGC formation in affected organs. In the ectopic GC, Tfh2 cells directly interact with B cells and, therefore, induce their differentiation from naïve B cells into plasmablasts and plasma cells capable of IgG4 secretion. Cytokines released by Tfh2, such as IL-4 and IL-21, play a role in IgG4 class switch, affinity maturation, and somatic hypermutation. Other subsets of T cell immunity also participate in the pathogenesis of IgG4-RD, resulting in IgG4-related tissue damage and fibrosis. APC, antigen presenting cell; Tfh2, T follicular helper 2 cells; Tfh1, T follicular helper 1 cell; FDC, follicular dendritic cell; CD40L, CD40 ligand. Created with BioRender.com.

Collectively, Tfh cells and their associated cytokines play a crucial role in the pathophysiology of IgG4-RD, exerting influence at every stage of disease progression, ranging from GC formation and plasmablast differentiation to the class switch of IgG4 antibodies. (**Figure 2**) A better understanding of their participation in IgG4-RD may provide novel therapeutic strategies targeting Tfh cells and Tfh-related cytokines against disease progression.

### 2.5 Circulating Tfh cells and correlations with disease activity

Circulating Tfh cells, exhibiting similar phenotypes and functions to Tfh cells, exist in blood circulation, which can be accessed within peripheral blood (11, 27). Circulating Tfh1, Tfh2 and Tfh17 are three major subsets of cTfh population (12, 13). Each subset has distinct cytokine profiles and functions in B cell differentiation, suggesting their respective pathogenic roles in IgG4-RD (68). Both cTfh1 and cTfh2 cells play an essential role in promoting B cell proliferation, whereas especially cTfh2 cells contribute to the differentiation of B cells into IgG4-producing plasma cells. In addition to Tfh cells, another subset known as follicular regulatory (Tfr) T cells has recently been identified to play a role in the pathogenesis of IgG4-RD.

Evaluation of disease activity holds significant value in early intervention to prevent irreversible organ fibrosis in IgG4-RD. Numerous studies have consistently demonstrated a positive correlation between cTfh and disease activity, as evidenced by the notable increase in Tfh cell count, particularly Tfh2 cells, among patients with IgG4-RD (16, 25, 69). Therefore, considering cTfh as a potential biomarker for monitoring disease activity in IgG4-RD shows promising prospects (11, 70).

#### 2.5.1 Tfh2 cells

Recent studies have demonstrated a significant increase in the number of circulating Tfh2 cells in patients with IgG4-RD compared with healthy controls (16, 55). Meanwhile, unlike Tfh1 cells, the number of Tfh2 cells was reported to be positively associated with the serum levels of IgG4 and the IgG4:IgG ratio (55, 71). Furthermore, a positive correlation was also observed between cTfh2 counts and the number of organs involved, implying their potential as indicators of disease activity and progression (55, 59, 71). Moreover, the number of activated cTfh2 cells in patients exhibited a decrease following glucocorticoid treatment yet demonstrated an increase during disease relapse (69, 71). Therefore, the number of activated cTfh2 cells may serve as a potential biomarker for monitoring disease activity and predicting relapse. These findings demonstrate that Tfh2 cells play a key role in plasmablast differentiation and IgG4 class switch, highlighting its involvement in the pathogenesis of IgG4-RD.

#### 2.5.2 Tfh1 cells

According to recent studies, the number of circulating activated Tfh1 cells is elevated in IgG4-RD (71). Tfh1 cells possess the ability to secrete IFN-γ but exhibit limited class-switching activity compared with the other two subsets. The quantity of Tfh1 cells exhibits a correlation with the disease activity of IgG4-RD. However, it does not vary with serum IgG4 levels. Therefore, it is inferred that Tfh1 cells may be involved in the pathogenesis of IgG4-RD but not in the production of IgG4 antibodies (71). On the other hand, another research extended these findings, showing that the activated SLAMF7^+^ cTfh1 cells may still affect IgG4 levels in patients by facilitating plasma cell differentiation (59). Therefore, further discussion is warranted regarding the role of Tfh1 cells in IgG4-RD.

#### 2.5.3 Tfh17 cells

Despite the active involvement of Tfh1 and Tfh2 in the pathogenesis of IgG4-RD, no evidence has yet reported the activation of Tfh17 cells in IgG4-RD. According to current knowledge, there appears to be no synchronous variation between the number of Tfh17 cells and serum IgG4 levels or the number of circulating plasmablasts (59, 71). Similarly, IL-17 expression, a hallmark cytokine produced by Tfh17 cells, is rarely detected in patients with IgG4-RD (34).

#### 2.5.4 Tfr cells

Tfr cells, a subset of CD4^+^T cells expressing CXCR5, have been demonstrated to collaborate with Tfh cells in the formation of GCs and Ig class switch (72). Tfr cells produce IL-10 and TGF-β, which affect the function of Tfh and B cells and contribute to the formation of GCs (73). Recent studies have demonstrated a significant increase in the number of circulating and tissue-infiltrating Tfr cells in patients with IgG4-RD. In addition, the proportion of Tfr cells is positively correlated with serum IgG4 levels and the number of organs involved, which suggests their potential involvement in the pathogenesis of IgG4-RD (74). Of note, compared with healthy controls, the number of IL-10-expressing circulating Tfr cells in patients with IgG4-RD was significantly increased. Considering the increased expression of IL-10 in affected tissues and its association with IgG4 class-switch, these findings suggest that Tfr cells may contribute to the class-switch recombination of IgG4 (74). Further investigation into the role of Tfr cells in IgG4-RD could provide valuable insight into their pathogenic mechanism.

### 2.6 Tph cells and their relationship with Tfh in IgG4-RD pathogenesis

T peripheral helper (Tph) cells (PD-1^hi^CXCR5^-^CD4^+^) are another subset of CD4^+^ T cells recently found to be involved in IgG4-RD development. Similar to Tfh cells, Tph cells can provide help for B cells via IL-21 and CXCL13 production. However, Tph cells express high levels of Blimp1 instead of Bcl6, and their unique expression of chemokine receptors, such as CCR2, CCR5, and CX3CR1, are correlated to their migration to the inflamed tissue (75). According to recent studies, the proportion of Tph cells was detected to be increased in IgG4-RD patients, positively associated with IgG4 serum concentrations as well as the number of organs involved. Additionally, their number experienced a decrease during clinical remission induced by glucocorticoids (60)..

According to recent findings, infiltration of Tph cells in affected lesions of IgG4-RD can be induced by their elevated expression of chemokine receptors. It is suggested that Tph can produce CXCL13, which triggers recruitment and accumulation of Tfh cells and B cells with the expression of CXCR5, leading to the formation of ectopic lymphoid structures and IgG4 production (76). Meanwhile, Tph cells also play a role as CD4^+^ CTLs by producing cytotoxic granules of GZMA, therefore participating in cytotoxic activity as well as destructive inflammation in affected lesions (60, 77). Results of one research indicated that CX3CR1^+^ Tph cells, rather than CX3CR1^-^ Tph cells, have a cytotoxic potential as CD4^+^ CTLs in IgG4-RD (77). Therefore, Tph cells are considered to contribute to persistent tissue injury in IgG4-RD and more insight into Tph cells as therapeutic targets for inhibiting IgG4-RD progression will be of great significance.

### 2.7 Cytotoxic Tfh cells infiltration of IgG4-RD and their correlations with CD4^+^CTLs

Cytotoxic Tfh cells are a subset of Tfh that has been novelly detected to infiltrate affected organs of IgG4-RD patients. CD4^+^CTLs were previously identified to be increased in circulation and affected lesions in patients with IgG4-RD, which contributed to cell apoptosis and tissue fibrosis. A comparative study recently has examined genes of cytotoxic molecules shared by cytotoxic Tfh cells and CD4^+^CTLs in IgG4-RD, including CRTAM, SLAMF7, NKG7, GZMA, GZMK, CCL4 and CCL5 (78). Meanwhile, it is also demonstrated that about 19 percent of CD4^+^CXCR5^+^Tfh cells expressed GZMK in one case of IgG4-RD. Evidence from nuclear distance measurements indicated that CD4^+^CXCR5^+^GXMK^+^Tfh cells were in physical contact with CD19^+^B cells (61). Additionally, GZMK^+^ cytotoxic Tfh cells can express fibrotic-related genes and therefore may correlate with tissue fibrosis (78). A thorough investigation will be required to elucidate their potential roles in IgG4-RD pathogenesis.

### 2.8 Collaborations of T cell subsets in the pathogenesis of IgG4-RD

In fact, the pathogenesis of IgG4-RD involves intricate immunological mechanisms, wherein diverse T cell subsets collaborate and participate in tissue damage and organ fibrosis. It is indicated that chronic exposure to a variety of unknown antigens triggers the activation of T cell immunity. Naïve T cells undergo differentiation and activation before evolving into multiple subsets with distinctive functions.

Tfh cells play a pivotal role in the pathogenesis of IgG4-RD, encompassing ectopic GC formation, IgG4 class switch and affinity maturation. They provide essential signals for B cell differentiation and release cytokines, including IL-4 and IL-21, which enable the differentiation of naïve B cells into plasmablasts and IgG4-secreting plasma cells, thereby augmenting IgG4 secretion. Subsequently, plasmablasts can migrate into inflamed tissues, where they participate in fibroblast activation and collagen production. IgG4 antibodies are characterized by their intrinsic anti-inflammatory properties and the ability to form immune complexes. It is suspected that they may potentially synergize with CTLs in promoting tissue inflammation and leading to organ damage.

Other subsets of T cell immunity, especially Treg, CTL and Th cells, also actively participate in IgG4-RD pathogenesis. There is an observed increase in the number of Treg cells both in circulation and affected tissues in IgG4-RD, suggesting their significant roles as key contributors to IgG4 class-switching and fibrosis (79, 80). Treg cells can express cytokines such as IL-10 and therefore participate in the differentiation of B cells into IgG4-producing plasmablasts and plasma cells, as well as facilitating IgG4 class-switching (81). Furthermore, it has been reported that the expression of the fibrogenic factor TGF-β is increased in fibrosis lesions of IgG4-RD, which indicates a potential association between Treg cells and organ fibrosis through TGF-β secretion (82).

In terms of tissue damage, CD4 and CD8 CTLs are major players. They produce granzyme, perforin, TGF-β and IL-1β, leading to cytolytic and profibrotic effects. Recently, accumulating experimental evidence has supported the involvement of CTLs in IgG4-RD, suggesting their capacity to induce tissue inflammation and fibrosis (83–85). Moreover, a recent study demonstrated the synergistic effect between CTLs and IgG4 antibodies in promoting tissue inflammation in IgG4-RD (86). Cytotoxic Tfh cells can express fibrotic-related genes similar to CD4^+^CTL and may also contribute to tissue inflammation and fibrosis.

**Figure 3:**
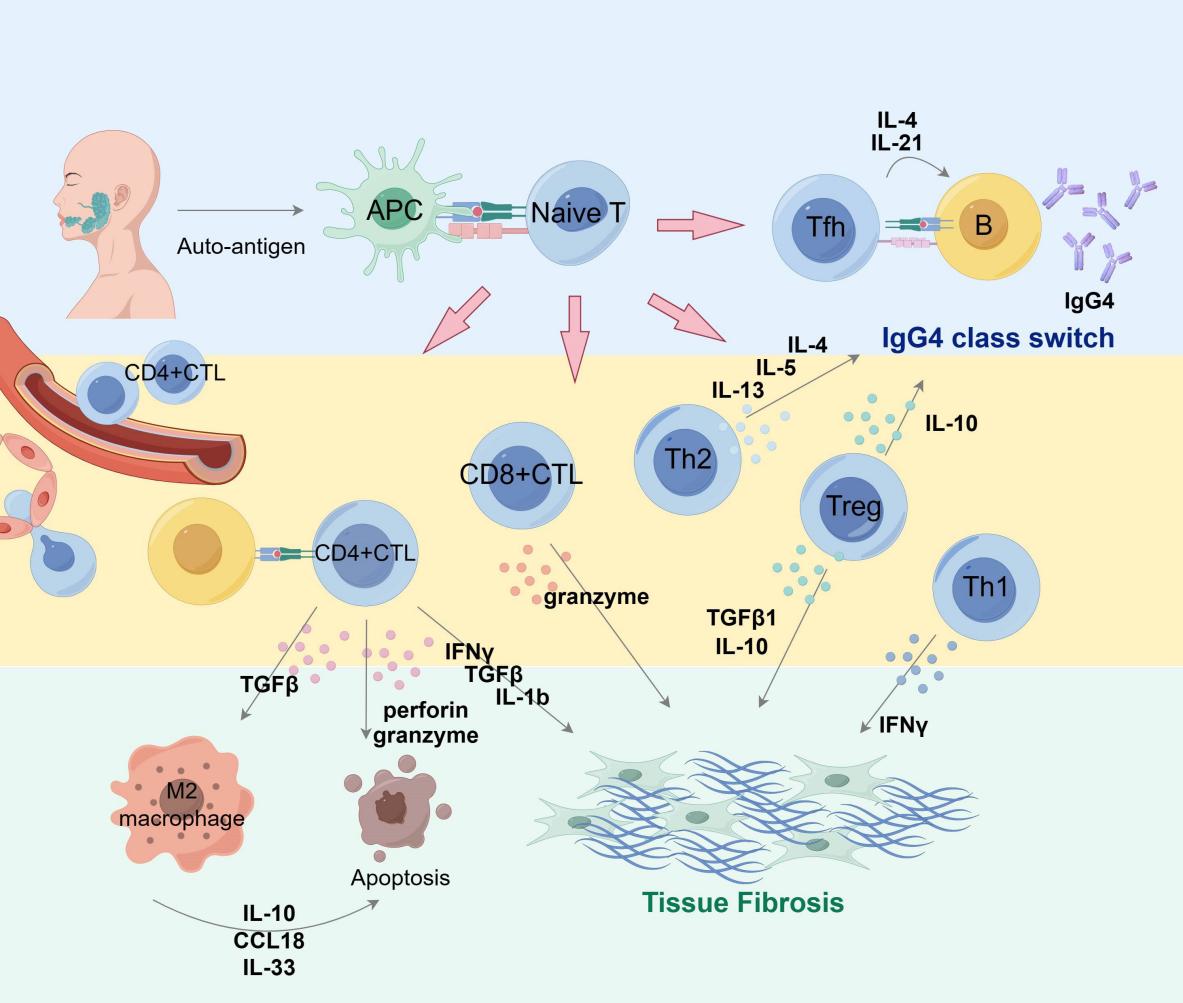
Overview of T cell subset collaboration in IgG4 production and fibrosis in pathogenesis of IgG4-RD. Antigen-presenting cells can present auto-antigen that stimulates T cells and induces their differentiation into multiple subsets of diverse properties and functions. Tfh cells interact with B cells to assist their maturation into IgG4-producing plasma cells and promote class switching via IL-4 and IL-21. Cytokines such as IL-10 and IL-13 are conducive factors in IgG4 class switch. CD4^+^ CTLs, CD8^+^CTLs and Treg cells actively participate in IgG4-related fibrosis via cytokine secretion. M2 macrophages also play a role in fibrosis formation. APC, antigen presenting cell; Tfh, follicular helper T cells. By Figdraw.

Th1 and Th2 are the two types of helper T cells initially described in history, and their responses are also suggested to be involved in T and B lymphocyte interaction in the ectopic germinal center, which proved to exert synergistic effects in progression of IgG4-RD (87). Researchers have found that Th1 populations were significantly increased in IgG4-RD, including the substantially elevated serum level of IFN-γ, which can activate Th1 cells (88, 89). The number of Th2 cells and cytokines secreted by them such as IL-4, IL-5 and IL-13 are also much higher at affected sites (85, 90). However, discrepancies exist regarding whether allergic factors related to Th2 are important in IgG4-RD development. (**Figure 3**, **Table 1**).

**Table 1:** Summary of pathogenic roles of T cell subsets in IgG4-related disease development.

T cell subsets	Involvement in the pathogenesis of IgG4-RD	Reference
**Th1 cells**	Increased Th1 population and production of IFN-γ, TGF-β	(88, 89)
**Th2 cells**	Increased number in affected tissue Increased production of IL-4, IL-5, IL-13	(85, 90) (85)
**CD4^+^ CTLs**	Increased number of circulating CD4^+^ CTLs Granzyme, TGF-β, and IL-1β production involved in fibrosis	(59) (83, 84)
**CD8^+^ CTLs**	Increased circulating CD8+ CTLs and granzyme production	(91)
**Treg cells**	Increased circulating Treg cells Associated with tissue fibrosis via IL-10, TGF-β production	(79, 80) (81, 82)
**Tfh cells**	Increased expression of CXCL13, BCL6, IL-21, CD40L Increased number of circulating Tfh cells Associated with IgG production	(18, 22, 27, 34) (16, 25, 69) (16, 25, 69)
**Tfh1 cells**	Increased circulating Tfh1 cells Associated with disease activity	(71) (71)
**Tfh2 cells**	Increased circulating Tfh2 cells Associated with disease activity Associated with IL-4, IL-21 production	(16, 55) (71) (38, 62, 63)

## 3 Therapy

### 3.1 Established therapeutic interventions in IgG4-RD

Glucocorticoids are currently the first-line therapy in patients with IgG4-RD, as recommended by the international consensus guidance established in 2015. However, there is still ongoing debate regarding optimal dose and duration (92). Studies have shown that glucocorticoids can induce disease remission by reducing levels of circulating activated Tfh2 cells and plasmablasts (93). Randomized controlled trials have been conducted to clarify the optimal dosage and necessity of maintenance glucocorticoid therapy (94, 95). However, due to the high incidence of drug resistance and relapse observed in clinical practice, particularly when tapering to low doses, prolonged courses of corticosteroids are often required for maintaining remission at the expense of increased glucocorticoid toxicity (38, 96, 97). According to collected data, only 5.7% of patients have achieved glucocorticoid discontinuation in routine clinical practice. Therefore, new therapeutic alternatives to glucocorticoids are appealing to optimize long-term intervention for patients with IgG4-RD.

Recent retrospective and prospective studies have shown that the combination of glucocorticoids and conventional immunosuppressants, such as cyclophosphamide and mycophenolate mofetil, is more efficacious than glucocorticoids alone in preventing relapse of IgG4-RD (98, 99). Nonetheless, the limitations of conventional steroid-sparing agents are evident. To date, there is a lack of prospective randomized controlled trials involving a significant number of patients. On the other hand, sustained immunosuppressive treatments may lead to extended toxicity exposure in IgG4-RD patients. Overall, the efficacy of immunosuppressive agents in achieving long-term steroid-free treatment with a safe profile remains suboptimal for patients with IgG4-RD. The contemporary situation highlights the priority to discover novel, safe, and effective therapeutic strategies.

Consequently, to enhance efficacy and reduce steroid-induced toxicity, molecular-targeted therapies have emerged as effective alternatives for glucocorticoid replacement therapy. The B-cell depletion with rituximab (RTX) targets CD20-positive B cells, which also depletes CD20-positive plasmablasts. Numerous studies have shown that RTX appears to be clinically effective, with a relatively high rate of treatment responses in IgG4-RD patients (100–102). However, it remains controversial whether RTX reduces the number of cTfh, as no significant change was observed in IgG4-RD patients after RTX treatment, according to some studies (103, 104). Additionally, challenges remain in terms of disease relapse and drug tolerance. In a prospective, open-label trial, over half of the IgG4-RD patients treated with rituximab experienced relapses. Adverse side events, including infections, were noted in patients after RTX treatment (101). Due to the lack of stable and effective alternatives, novel therapeutic strategies are required as glucocorticoid-sparing and maintenance therapy for IgG4-RD. Advances in understanding the pathophysiology and the pivotal function of Tfh in IgG4-RD will deeply contribute to the emergence of new targeted approaches in treating the disease.

### 3.2 Potential therapeutic strategies targeting Tfh cells

**Figure 4:**
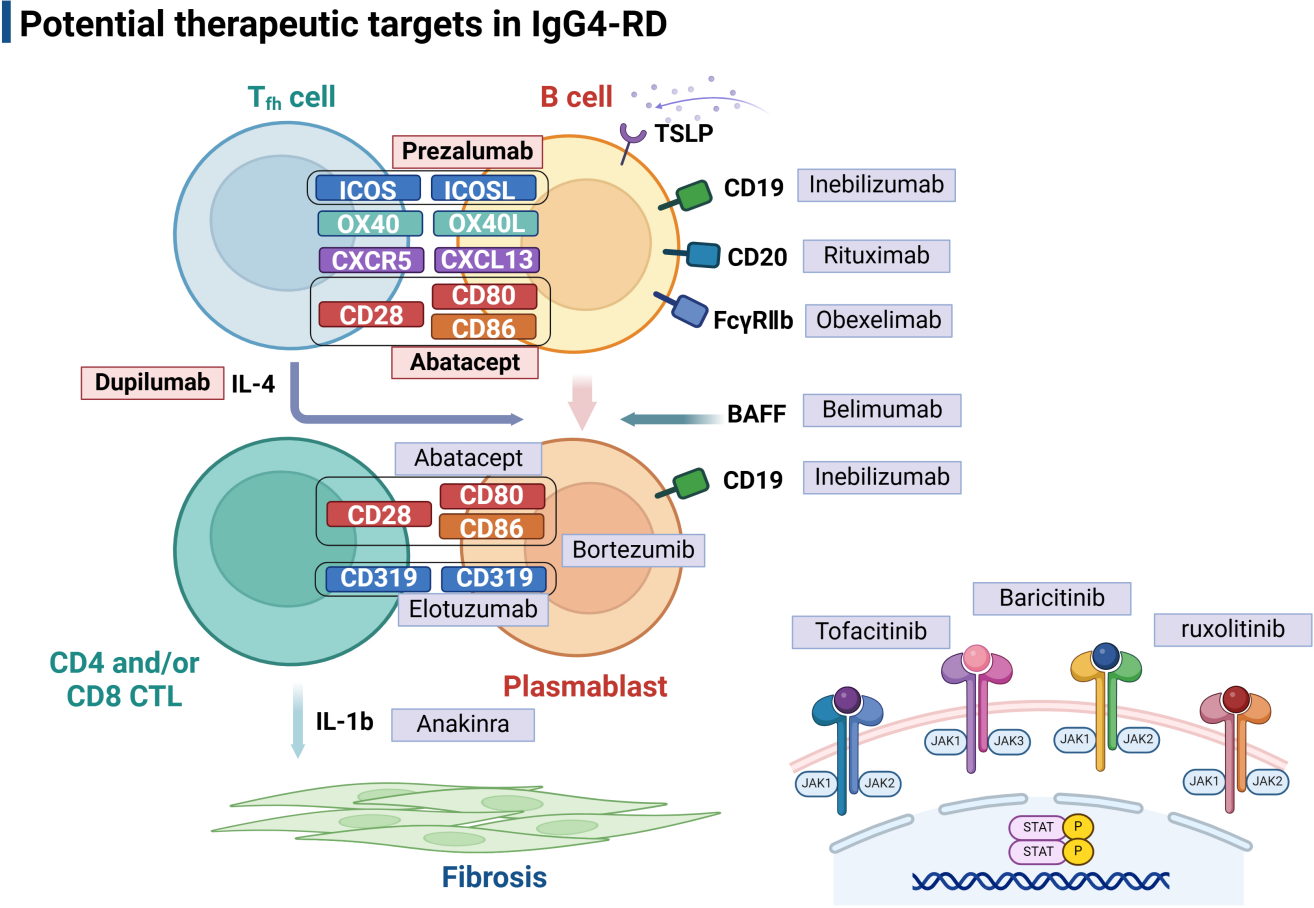
Potential therapeutic targets in IgG4-related disease. The picture illustrated Tfh-B interactions in IgG4-RD pathogenesis at different levels, and targeted therapies are used to interfere with these mechanisms at each level. Follicular T helper cells play a central role in assisting B cell differentiation into IgG4 secreting plasmablasts, which therefore also provide valuable targets for therapeutic intervention, including Abatacept and Prezalumab that inhibit B-T cell co-stimulation and Duplimab targeting Tfh-related cytokines. JAK inhibitors, including Tofacitinib, Baricitinib, and ruxolitinib, are potential therapeutic targets in IgG4-RD treatment. Tfh, follicular helper T cell; CTL, cytotoxic T cell; TSLP, Thymic stromal lymphopoietin. Created with BioRender.com.

The remarkable progress in molecular-targeted therapies in recent years has significantly advanced the treatment of autoimmune diseases such as rheumatoid arthritis(RA) and systemic lupus erythematosus(SLE), providing novel therapeutic options for IgG4-RD (105, 106). Recent studies have highlighted the importance of Tfh cells in the pathogenesis of IgG4-RD and their potential as therapeutic targets. Based on mechanistic understanding of the disease, we will briefly discuss several potential promising Tfh-related targets for treating IgG4-RD in the subsequent section. (**Figure 4**, **Table 2**).

**Table 2:** Emerging targeted monoclonal antibodies with potential therapeutic effects in IgG4-RD.

mAb	Target	Mechanisms	Current status	Reference
**Abatacept**	Tfh cell	CTLA-4 fusion protein with CD28 co-stimulation blockade	Case report	(107)
**Prezalumab**	Tfh cell	ICOSL-directed Tfh cell inhibition	Not studied	(108)
**Dupilumab**	Tfh-related cytokine	IL-4R/IL-13 signaling inhibition	Case report	(109)
**Tezepelumab**	Tfh-related cytokine	TSLP inhibition	Not studied	(110)
**Rituximab**	B cell	CD20^+^ B cell depletion	Clinical trial (completed)	(100)
**Inebilizumab**	B cell	CD19^+^ B cell depletion	Clinical trial (active not recruiting)	(111)
**Belimumab**	B cell	BAFF inhibition	Clinical trial (recruiting)	(112, 113)
**Obexelimab**	B cell	FcγRIIb/CD19 inhibition	Clinical trial (completed)	(114)
**Bortezomib**	B cell	Proteasome inhibition	Case report	(115)
**Elotuzumab**	CTL	SLAM-F7–directed ADCC activation	Clinical trial (terminated)	(116)
**Mepolizumab**	cytokine	IL-5/IL-5R axis inhibition	Case report	(117)

#### 3.2.1 Targeted Tfh-related surface molecules

##### 3.2.1.1 Targeting CD80/86 –Abatacept

Abatacept is a fusion protein consisting of the extracellular domain of cytotoxic T lymphocyte-associated antigen 4(CTLA4) and an IgG1 Fc fragment (118). T cells rely on both signals of TCR-mediated activation and CD28-mediated co-stimulation to activate and differentiate into effector forms, wherein CD28 on the surface of T cells binds with CD80/CD86 on antigen-presenting cells to produce a co-stimulation effect during this process (118). Abatacept exhibits higher binding affinity for CD80/CD86 and, therefore, can compete with ligation to CD28 on the T cell surface to prevent T cell activation (118). As one of the biologic DMARDs in RA therapy, abatacept reduces the number of Tfh cells and has also achieved favorable clinical response in patients with IgG4-RD (119, 120). It is suspected that in patients with IgG4-RD, abatacept functions mainly by inhibiting Tfh cells and disrupting ectopic GCs formation or directly depleting expanded CD80/86-expressing plasmablasts through Fc-mediated mechanisms (107, 121). Moreover, it has been shown that clinical responses of abatacept are associated with activated Tfh2 cells, circulating plasmablasts and serum IgE levels and can be predicted by baseline proportions of unswitched memory B cells (107). Nevertheless, further clarification regarding its safety and efficacy in a larger cohort of IgG4-RD cases is warranted before widespread clinical application.

##### 3.2.1.2 Targeting ICOSL –Prezalumab

As formerly mentioned, ICOS/ICOSL signaling is essential in sustaining Tfh cell differentiation, facilitating T-B interactions, promoting antibody production, and supporting GC formation (24). Therefore, the blockade of ICOSL could be an effective strategy for treating IgG4-RD. Prezalumab, a fully human IgG2a antibody targeting ICOSL, formerly known as AMG-557, has shown safety and potential efficacy in clinical trials of patients with Sjogren’s syndrome and SLE (108, 122, 123). Rozibafusp alfa (AMG 570), a bispecific inhibitory molecule targeting both ICOSL and B-cell activating factor BAFF, is currently undergoing phase I clinical trials and is expected to be more efficient in the treatment of autoimmune diseases such as SLE and RA (124, 125). In the future, it is promising to be applied for therapy in a broader spectrum of autoimmune diseases, including IgG4-RD.

##### 3.2.1.3 Other possible surface targets for IgG4-RD treatment

Several studies have indicated a positive correlation between upregulated OX40 signaling and autoimmune diseases which are attributed to aberrant Tfh activity (30). Therapeutic interventions targeting OX40/OX40L interaction may help regulate T-cell responses and provide novel solutions to T-cell-mediated autoimmune diseases such as IgG4-RD. Accumulating experiments have been conducted to verify the therapeutic efficacy of blocking OX40 or OX40L, such as anti-OX40 monoclonal antibodies. It is suggested that OX40/OX40L blockade inhibits the proliferation of active CD4^+^ T cells by preventing their migration, moderating their activation and cytokine production (126). A recent RNA-sequencing study identified a significant correlation between the OX40 signaling and high expression of IL-21 in IgG4-RD patients, indicating the possible contribution of the OX40 signaling in IL-21 production and the pathogenesis of IgG4-RD. Since the production of IL-21 by Tfh cells is associated with serum IgG4 level and disease activity, these findings imply that the OX40 signal inhibition could be a promising therapeutic approach (69).

#### 3.2.2 Targeted Tfh-related cytokines

##### 3.2.2.1 Targeting IL-4R and IL-13 –Dupilumab

Cytokine IL-4 plays a pivotal role in IgG4-RD and is closely related to IgG4 production and IgG4 class switch mediated by Tfh cells (3, 38). Dupilumab, a humanized IgG4 monoclonal antibody targeting the IL-4 receptor-α chain (IL-4Rα), exerts dual antagonistic effects on both IL-4 and IL-13 signaling pathways (127). It has already been approved for treating asthma, chronic rhinosinusitis with nasal polyposis, atopic dermatitis and other type 2 inflammatory diseases (128–130). Recently, several case reports have demonstrated that dupilumab can ameliorate the disease activity and may serve as a steroid-sparing agent in patients with IgG4-RD (109, 131, 132). In addition, one report indicates a reduction in circulating Tfh cell count (133), which explains the ability of dupilumab to suppress the serum IgG4 levels and IgG4-mediated inflammation. Despite the existence of limited case reports, no definitive conclusions can be drawn regarding the efficacy of dupilumab in treating IgG4-RD. However, given the central role of IL-4 and IL-13 in IgG4 class switch and tissue fibrosis, treatment targeting these pathways in IgG4-RD remains promising. Further exploration through randomized clinical trials is warranted to confirm its efficacy.

##### 3.2.2.2 Targeting TSLP –Tezepelumab

Thymic stromal lymphopoietin (TSLP), a cytokine released by a variety of cells, such as epithelial cells and fibroblasts, plays a vital role in maintaining immune homeostasis and type 2 immune responses (134). Dysregulation of TSLP expression is closely related to immunopathology in several diseases, including RA and systemic sclerosis (SSc) (135). Recent studies have shown elevated TSLP expression in IgG4-RD, suggesting its involvement in the pathogenesis of this condition (136). TSLP activates the JAK-STAT signaling pathway and promotes B cell proliferation and IgG4 antibody secretion by acting with receptors TSLPR and IL7Ra on B cells. By upregulating the expression of OX40L, TSLP-activated B cells induce the polarization of CD4^+^ naïve T cells into Tfh cells that are essential in IgG4-RD pathophysiology (137). Results from emerging clinical trials of anti-TSLP agents have already confirmed the efficacy of treatment in many inflammatory conditions, such as asthma. For instance, recently, Tezepelumab, a human IgG2 monoclonal antibody that inhibits the binding of TSLP to its receptor, has been undergoing clinical trials for asthma treatment (110, 138). Therefore, potential therapeutic strategies targeting the TSLP-TSLP receptor axis may also provide new solutions to IgG4-RD treatment.

##### 3.2.2.3 Other possible cytokine targets for IgG4-RD treatment

In IgG4-RD, expressions of CXCL13 and CXCR5 are elevated, and CXCR5-CXCL13 interaction provides signals for Tfh migration and interaction with B cells in GC (139). Recent findings have suggested the pathogenic roles of the CXCL13/CXCR5 axis in autoimmune diseases, which indicates the potential role of CXCL13 as a disease biomarker and therapeutic target (140). Meanwhile, according to a study conducted on autoimmune pancreatitis (AIP), a common feature in IgG4-RD, it is observed that LTα and LTβ are overexpressed and positively correlate with CXCL13 expression. Through inhibiting LTβR-signaling, disease features can be significantly alleviated, providing new signaling targets for therapeutic strategies (139).

#### 3.2.3 Targeting intracellular signaling pathways

The Janus kinase (JAK) family of intracellular, non-receptor tyrosine kinases is associated with effects of a wide variety of key cytokines. The JAK-STAT pathway is an important signal transducer implicated in immune dysregulation, making it an essential therapeutic target for autoimmune and inflammatory diseases (141). Recently, researchers also have predicted JAK inhibitors might play a significant role in treatment of IgG4-RD based on the complex cytokine network in immune regulation (142). Similar to clinical trials conducted in other autoimmune diseases with promising results, these inhibitors may exhibit potent effects in preventing tissue fibrosis and ameliorating inflammation among patients with IgG4-RD. Tofacitinib, a JAK1/3 inhibitor, can effectively control fibrosis and tissue inflammation by inhibiting synovial fibroblast migration and down-regulating inflammatory cytokine production (143). Through its inhibition of Tfh induction, Tofacitinib can reduce pancreatic inflammation in IgG4-RD (86). Furthermore, since the account of circulating CD28^-^ CTLs increased in patients with IgG4-RD, tofacitinib inhibits IL-2 signaling, which induces CTL proteome and effector molecules, indicating that CTLs could be a potential target for the treatment of IgG4-RD with this drug. Meanwhile, another study found that IL-7 extensively promotes expansion and function of CTLs through activation of the JAK pathway (144). Accordingly, JAK inhibitors may exert a therapeutic effect by blocking the IL-7 signaling pathway, which is a promising strategy worth exploring. Baricitinib, a JAK 1/2 inhibitor, may become an ideal candidate for managing IgG4-RD by inhibiting CD80/CD86 expression and INF-1 production of dendritic cells, including IL-6 production and B cell differentiation. Other JAK inhibitors, such as ruxolitinib and itacitinib, are also promising as they can inhibit IL-4 and IL-13 signaling, which has been shown to be effective in alleviating fibrosis and tissue inflammation (145, 146).

## 4 Conclusions and perspectives

Current evidence suggests the involvement of Tfh cells in IgG4-RD. It is concluded that the participation of Tfh subsets may play a crucial role in ectopic GC formation in affected tissues as well as differentiation of B cells into IgG4-producing plasmablasts, leading to abnormal IgG4 production and irreversible tissue damage. Targets at signals required for Tfh cell development or function, along with cytokines, could potentially reduce the proportion of Tfh cells or modulate their functions in GC responses to control IgG4-RD development. A better understanding of the pathogenic roles of Tfh cells will pave the way for advances in novel targeted immunotherapies for IgG4-RD patients. Future research will focus on conducting more clinical trials for novel biologically targeted treatment in exploration of the optimal scheme for clinical intervention. This will provide guidance in improving long-term outcomes of IgG4-RD patients.

## Author contributions

JX: Writing – original draft. JYZ: Writing – review & editing. JXZ: Writing – review & editing.

## References

[1] Kamisawa T Zen Y Pillai S Stone JH. IgG4-related disease. Lancet. (2015) 385:1460–71. doi: 10.1016/S0140-6736(14)60720-0 25481618

[2] Lanzillotta M Mancuso G Della-Torre E. Advances in the diagnosis and management of IgG4 related disease. Bmj. (2020) 369:m1067. doi: 10.1136/bmj.m1067 32546500

[3] Perugino CA Stone JH. IgG4-related disease: an update on pathophysiology and implications for clinical care. Nat Rev Rheumatol. (2020) 16:702–14. doi: 10.1038/s41584-020-0500-7 32939060

[4] Kamisawa T Egawa N Nakajima H. Autoimmune pancreatitis is a systemic autoimmune disease. Am J Gastroenterol. (2003) 98:2811–2. doi: 10.1111/j.1572-0241.2003.08758.x 14687846

[5] Kamisawa T Funata N Hayashi Y Eishi Y Koike M Tsuruta K. A new clinicopathological entity of IgG4-related autoimmune disease. J Gastroenterol. (2003) 38:982–4. doi: 10.1007/s00535-003-1175-y 14614606

[6] Liu J Yin W Westerberg LS Lee P Gong Q Chen Y. Immune dysregulation in igG(4)-related disease. Front Immunol. (2021) 12:738540. doi: 10.3389/fimmu.2021.738540 34539675 PMC8440903

[7] Crotty S. T follicular helper cell differentiation, function, and roles in disease. Immunity. (2014) 41:529–42. doi: 10.1016/j.immuni.2014.10.004 PMC4223692 25367570

[8] Ma CS Deenick EK. Human T follicular helper (Tfh) cells and disease. Immunol Cell Biol. (2014) 92:64–71. doi: 10.1038/icb.2013.55 24145858

[9] Ji LS Sun XH Zhang X Zhou ZH Yu Z Zhu XJ. Mechanism of follicular helper T cell differentiation regulated by transcription factors. J Immunol Res. (2020) 2020:1826587. doi: 10.1155/2020/1826587 32766317 PMC7387970

[10] Crotty S. Follicular helper CD4 T cells (TFH). Annu Rev Immunol. (2011) 29:621–63. doi: 10.1146/annurev-immunol-031210-101400 21314428

[11] Ueno H. Human circulating T follicular helper cell subsets in health and disease. J Clin Immunol. (2016) 36 Suppl 1:34–9. doi: 10.1007/s10875-016-0268-3 26984851

[12] Morita R Schmitt N Bentebibel SE Ranganathan R Bourdery L Zurawski G. Human blood CXCR5(+)CD4(+) T cells are counterparts of T follicular cells and contain specific subsets that differentially support antibody secretion. Immunity. (2011) 34:108–21. doi: 10.1016/j.immuni.2010.12.012 PMC3046815 21215658

[13] Kasashima S Kawashima A Kurose N Ozaki S Ikeda H Harada K. The disturbance of the distribution of T helper cell subsets in the mantle area surrounding germinal centers in immunoglobulin G4-related sclerosing sialadenitis. Virchows Arch. (2022) 481:767–77. doi: 10.1007/s00428-022-03384-7 35902401

[14] Mintz MA Cyster JG. T follicular helper cells in germinal center B cell selection and lymphomagenesis. Immunol Rev. (2020) 296:48–61. doi: 10.1111/imr.12860 32412663 PMC7817257

[15] Kamekura R Takano K Yamamoto M Kawata K Shigehara K Jitsukawa S. Cutting edge: A critical role of lesional T follicular helper cells in the pathogenesis of igG4-related disease. J Immunol. (2017) 199:2624–9. doi: 10.4049/jimmunol.1601507 28916523

[16] Grados A Ebbo M Piperoglou C Groh M Regent A Samson M. T cell polarization toward T(H)2/T(FH)2 and T(H)17/T(FH)17 in patients with igG4-related disease. Front Immunol. (2017) 8:235. doi: 10.3389/fimmu.2017.00235 28348556 PMC5347096

[17] Crotty S. T follicular helper cell biology: A decade of discovery and diseases. Immunity. (2019) 50:1132–48. doi: 10.1016/j.immuni.2019.04.011 PMC6532429 31117010

[18] Kudo-Tanaka E Nakatsuka S Hirano T Kawai M Katada Y Matsushita M. A case of Mikulicz's disease with Th2-biased cytokine profile: possible feature discriminable from Sjögren's syndrome. Mod Rheumatol. (2009) 19:691–5. doi: 10.1007/s10165-009-0214-9 19697095

[19] Ohta M Moriyama M Maehara T Gion Y Furukawa S Tanaka A. DNA microarray analysis of submandibular glands in igG4-related disease indicates a role for MARCO and other innate immune-related proteins. Med (Baltimore). (2016) 95:e2853. doi: 10.1097/MD.0000000000002853 PMC4998650 26886650

[20] Jogdand GM Mohanty S Devadas S. Regulators of tfh cell differentiation. Front Immunol. (2016) 7:520. doi: 10.3389/fimmu.2016.00520 27933060 PMC5120123

[21] Breitfeld D Ohl L Kremmer E Ellwart J Sallusto F Lipp M. Follicular B helper T cells express CXC chemokine receptor 5, localize to B cell follicles, and support immunoglobulin production. J Exp Med. (2000) 192:1545–52. doi: 10.1084/jem.192.11.1545 PMC2193094 11104797

[22] Esposito I Born D Bergmann F Longerich T Welsch T Giese NA. Autoimmune pancreatocholangitis, non-autoimmune pancreatitis and primary sclerosing cholangitis: a comparative morphological and immunological analysis. PLoS One. (2008) 3:e2539. doi: 10.1371/journal.pone.0002539 18596913 PMC2440515

[23] Havenar-Daughton C Lindqvist M Heit A Wu JE Reiss SM Kendric K. CXCL13 is a plasma biomarker of germinal center activity. Proc Natl Acad Sci U.S.A. (2016) 113:2702–7. doi: 10.1073/pnas.1520112113 PMC4790995 26908875

[24] Akiba H Takeda K Kojima Y Usui Y Harada N Yamazaki T. The role of ICOS in the CXCR5+ follicular B helper T cell maintenance in vivo. J Immunol. (2005) 175:2340–8. doi: 10.4049/jimmunol.175.4.2340 16081804

[25] Cargill T Makuch M Sadler R Lighaam LC Peters R van Ham M. Activated T-follicular helper 2 cells are associated with disease activity in igG4-related sclerosing cholangitis and pancreatitis. Clin Transl Gastroenterol. (2019) 10:e00020. doi: 10.14309/ctg.0000000000000020 31033594 PMC6602789

[26] Zhang X Lu H Peng L Zhou J Wang M Li J. The role of PD-1/PD-Ls in the pathogenesis of IgG4-related disease. Rheumatol (Oxford). (2022) 61:815–25. doi: 10.1093/rheumatology/keab360 33930105

[27] Chen Y Lin W Yang H Wang M Zhang P Feng R. Aberrant expansion and function of follicular helper T cell subsets in igG4-related disease. Arthritis Rheumatol. (2018) 70:1853–65. doi: 10.1002/art.40556 PMC6220938 29781221

[28] Vinuesa CG Linterman MA Goodnow CC Randall KL. T cells and follicular dendritic cells in germinal center B-cell formation and selection. Immunol Rev. (2010) 237:72–89. doi: 10.1111/j.1600-065X.2010.00937.x 20727030

[29] Kawabe T Naka T Yoshida K Tanaka T Fujiwara H Suematsu S. The immune responses in CD40-deficient mice: impaired immunoglobulin class switching and germinal center formation. Immunity. (1994) 1:167–78. doi: 10.1016/1074-7613(94)90095-7 7534202

[30] Fu N Xie F Sun Z Wang Q. The OX40/OX40L axis regulates T follicular helper cell differentiation: implications for autoimmune diseases. Front Immunol. (2021) 12:670637. doi: 10.3389/fimmu.2021.670637 34234777 PMC8256170

[31] Edner NM Carlesso G Rush JS Walker LSK. Targeting co-stimulatory molecules in autoimmune disease. Nat Rev Drug Discovery. (2020) 19:860–83. doi: 10.1038/s41573-020-0081-9 32939077

[32] Weinberg AD. OX40: targeted immunotherapy–implications for tempering autoimmunity and enhancing vaccines. Trends Immunol. (2002) 23:102–9. doi: 10.1016/S1471-4906(01)02127-5 11929124

[33] Johnston RJ Poholek AC DiToro D Yusuf I Eto D Barnett B. Bcl6 and Blimp-1 are reciprocal and antagonistic regulators of T follicular helper cell differentiation. Science. (2009) 325:1006–10. doi: 10.1126/science.1175870 PMC2766560 19608860

[34] Maehara T Moriyama M Nakashima H Miyake K Hayashida JN Tanaka A. Interleukin-21 contributes to germinal centre formation and immunoglobulin G4 production in IgG4-related dacryoadenitis and sialoadenitis, so-called Mikulicz's disease. Ann Rheum Dis. (2012) 71:2011–19. doi: 10.1136/annrheumdis-2012-201477 22753386

[35] Yang JA Tubo NJ Gearhart MD Bardwell VJ Jenkins MK. Cutting edge: Bcl6-interacting corepressor contributes to germinal center T follicular helper cell formation and B cell helper function. J Immunol. (2015) 194:5604–8. doi: 10.4049/jimmunol.1500201 PMC4458443 25964495

[36] Nurieva RI Podd A Chen Y Alekseev AM Yu M Qi X. STAT5 protein negatively regulates T follicular helper (Tfh) cell generation and function. J Biol Chem. (2012) 287:11234–9. doi: 10.1074/jbc.M111.324046 PMC3322890 22318729

[37] Betz BC Jordan-Williams KL Wang C Kang SG Liao J Logan MR. Batf coordinates multiple aspects of B and T cell function required for normal antibody responses. J Exp Med. (2010) 207:933–42. doi: 10.1084/jem.20091548 PMC2867277 20421391

[38] Maehara T Mattoo H Mahajan VS Murphy SJ Yuen GJ Ishiguro N. The expansion in lymphoid organs of IL-4(+) BATF(+) T follicular helper cells is linked to IgG4 class switching in vivo. Life Sci Alliance. (2018) 1(1):e201800050. doi: 10.1101/284737 29984361 PMC6034714

[39] Sahoo A Alekseev A Tanaka K Obertas L Lerman B Haymaker C. Batf is important for IL-4 expression in T follicular helper cells. Nat Commun. (2015) 6:7997. doi: 10.1038/ncomms8997 26278622 PMC4557271

[40] Mattoo H Mahajan VS Della-Torre E Sekigami Y Carruthers M Wallace ZS. De novo oligoclonal expansions of circulating plasmablasts in active and relapsing IgG4-related disease. J Allergy Clin Immunol. (2014) 134:679–87. doi: 10.1016/j.jaci.2014.03.034 PMC4149918 24815737

[41] Sato Y Kojima M Takata K Morito T Mizobuchi K Tanaka T. Multicentric Castleman's disease with abundant IgG4-positive cells: a clinical and pathological analysis of six cases. J Clin Pathol. (2010) 63:1084–9. doi: 10.1136/jcp.2010.082958 20974624

[42] Victora GD Nussenzweig MC. Germinal centers. Annu Rev Immunol. (2022) 40:413–42. doi: 10.1146/annurev-immunol-120419-022408 35113731

[43] Satoh-Nakamura T Kurose N Kawanami T Nakamura T Iwao-Kawanami H Nakajima A. CD14+ follicular dendritic cells in lymphoid follicles may play a role in the pathogenesis of IgG4-related disease. BioMed Res. (2015) 36:143–53. doi: 10.2220/biomedres.36.143 25876665

[44] Weinstein JS Herman EI Lainez B Licona-Limón P Esplugues E Flavell R. TFH cells progressively differentiate to regulate the germinal center response. Nat Immunol. (2016) 17:1197–205. doi: 10.1038/ni.3554 PMC5030190 27573866

[45] Zaidan M Cervera-Pierot P de Seigneux S Dahan K Fabiani B Callard P. Evidence of follicular T-cell implication in a case of IgG4-related systemic disease with interstitial nephritis. Nephrol Dial Transplant. (2011) 26:2047–50. doi: 10.1093/ndt/gfr097 21406542

[46] Koga R Maehara T Aoyagi R Munemura R Murakami Y Doi A. Granzyme K- and amphiregulin-expressing cytotoxic T cells and activated extrafollicular B cells are potential drivers of IgG4-related disease. J Allergy Clin Immunol. (2024) 153:1095–112. doi: 10.1016/j.jaci.2023.11.916 38092138

[47] Ruschil C Gabernet G Lepennetier G Heumos S Kaminski M Hracsko Z. Specific induction of double negative B cells during protective and pathogenic immune responses. Front Immunol. (2020) 11:606338. doi: 10.3389/fimmu.2020.606338 33391273 PMC7775384

[48] Morgan D Tergaonkar V. Unraveling B cell trajectories at single cell resolution. Trends Immunol. (2022) 43:210–29. doi: 10.1016/j.it.2022.01.003 35090788

[49] Allard-Chamard H Kaneko N Bertocchi A Sun N Boucau J Kuo HH. Extrafollicular IgD(-)CD27(-)CXCR5(-)CD11c(-) DN3 B cells infiltrate inflamed tissues in autoimmune fibrosis and in severe COVID-19. Cell Rep. (2023) 42:112630. doi: 10.1016/j.celrep.2023.112630 37300833 PMC10227203

[50] Rispens T Huijbers MG. The unique properties of IgG4 and its roles in health and disease. Nat Rev Immunol. (2023) 23:763–78. doi: 10.1038/s41577-023-00871-z PMC10123589 37095254

[51] Wallace ZS Mattoo H Carruthers M Mahajan VS Della Torre E Lee H. Plasmablasts as a biomarker for IgG4-related disease, independent of serum IgG4 concentrations. Ann Rheum Dis. (2015) 74:190–5. doi: 10.1136/annrheumdis-2014-205233 PMC4656194 24817416

[52] Wallace ZS Deshpande V Mattoo H Mahajan VS Kulikova M Pillai S. IgG4-related disease: Clinical and laboratory features in one hundred twenty-five patients. Arthritis Rheumatol. (2015) 67:2466–75. doi: 10.1002/art.39205 PMC4621270 25988916

[53] Lu C Li S Qing P Zhang Q Ji X Tang Z. Single-cell transcriptome analysis and protein profiling reveal broad immune system activation in IgG4-related disease. JCI Insight. (2023) 8(17):e167602. doi: 10.1172/jci.insight.167602 37561593 PMC10544205

[54] Della-Torre E Rigamonti E Perugino C Baghai-Sain S Sun N Kaneko N. B lymphocytes directly contribute to tissue fibrosis in patients with IgG(4)-related disease. J Allergy Clin Immunol. (2020) 145:968–981.e14. doi: 10.1016/j.jaci.2019.07.004 31319101 PMC6960365

[55] Akiyama M Suzuki K Yamaoka K Yasuoka H Takeshita M Kaneko Y. Number of circulating follicular helper 2 T cells correlates with igG4 and interleukin-4 levels and plasmablast numbers in igG4-related disease. Arthritis Rheumatol. (2015) 67:2476–81. doi: 10.1002/art.39209 25989153

[56] Kubo S Nakayamada S Zhao J Yoshikawa M Miyazaki Y Nawata A. Correlation of T follicular helper cells and plasmablasts with the development of organ involvement in patients with IgG4-related disease. Rheumatol (Oxford). (2018) 57:514–24. doi: 10.1093/rheumatology/kex455 29253269

[57] Lin W Zhang P Chen H Chen Y Yang H Zheng W. Circulating plasmablasts/plasma cells: a potential biomarker for IgG4-related disease. Arthritis Res Ther. (2017) 19:25. doi: 10.1186/s13075-017-1231-2 28183334 PMC5301376

[58] Wang Y Zhao Z Gao D Wang H Liao S Luo G. Clinical value of plasmablasts in predicting disease relapse in patients with IgG4-related disease. Clin Rheumatol. (2023) 42:135–43. doi: 10.1007/s10067-022-06339-0 36074221

[59] Higashioka K Ota Y Maehara T Moriyama M Ayano M Mitoma H. Association of circulating SLAMF7(+)Tfh1 cells with IgG4 levels in patients with IgG4-related disease. BMC Immunol. (2020) 21:31. doi: 10.1186/s12865-020-00361-0 32487061 PMC7268355

[60] Kamekura R Yamamoto M Takano K Yabe H Ito F Ikegami I. Circulating PD-1(+)CXCR5(-)CD4(+) T cells underlying the immunological mechanisms of IgG4-related disease. Rheumatol Adv Pract. (2018) 2:rky043. doi: 10.1093/rap/rky043 31431980 PMC6649940

[61] Munemura R Maehara T Murakami Y Koga R Aoyagi R Kaneko N. Distinct disease-specific Tfh cell populations in 2 different fibrotic diseases: IgG(4)-related disease and Kimura disease. J Allergy Clin Immunol. (2022) 150:440–455.e17. doi: 10.1016/j.jaci.2022.03.034 35568079 PMC10369367

[62] Sasaki T Akiyama M Kaneko Y Takeuchi T. Immunoglobulin G4-related disease and idiopathic multicentric Castleman's disease: confusable immune-mediated disorders. Rheumatol (Oxford). (2022) 61:490–501. doi: 10.1093/rheumatology/keab634 34363463

[63] Akiyama M Yasuoka H Yoshimoto K Takeuchi T. Interleukin-4 contributes to the shift of balance of IgG subclasses toward IgG4 in IgG4-related disease. Cytokine. (2018) 110:416–9. doi: 10.1016/j.cyto.2018.05.009 29861381

[64] Shankar A McAlees JW Lewkowich IP. Modulation of IL-4/IL-13 cytokine signaling in the context of allergic disease. J Allergy Clin Immunol. (2022) 150:266–76. doi: 10.1016/j.jaci.2022.06.012 PMC9371363 35934680

[65] Ettinger R Sims GP Fairhurst AM Robbins R da Silva YS Spolski R. IL-21 induces differentiation of human naive and memory B cells into antibody-secreting plasma cells. J Immunol. (2005) 175:7867–79. doi: 10.4049/jimmunol.175.12.7867 16339522

[66] Bessa J Kopf M Bachmann MF. Cutting edge: IL-21 and TLR signaling regulate germinal center responses in a B cell-intrinsic manner. J Immunol. (2010) 184:4615–9. doi: 10.4049/jimmunol.0903949 20368279

[67] Yajima H Yamamoto M Shimizu Y Sakurai N Suzuki C Naishiro Y. Loss of interleukin-21 leads to atrophic germinal centers in multicentric Castleman's disease. Ann Hematol. (2016) 95:35–40. doi: 10.1007/s00277-015-2500-2 26377996

[68] Murayama K Ikegami I Kamekura R Sakamoto H Yanagi M Kamiya S. CD4(+)CD8(+) T follicular helper cells regulate humoral immunity in chronic inflammatory lesions. Front Immunol. (2022) 13:941385. doi: 10.3389/fimmu.2022.941385 36091071 PMC9452889

[69] Akiyama M Suzuki K Yoshimoto K Yasuoka H Kaneko Y Takeuchi T. Peripheral TIGIT+ T follicular helper cells that produce high levels of interleukin-21 via OX40 represent disease activity in igG4-related disease. Front Immunol. (2021) 12:651357. doi: 10.3389/fimmu.2021.651357 33936071 PMC8079782

[70] Han L Yang X Yu Y Wan W Lv L Zou H. Associations of circulating CXCR3(-)PD-1(+)CD4(+)T cells with disease activity of systemic lupus erythematosus. Mod Rheumatol. (2019) 29:461–9. doi: 10.1080/14397595.2018.1469581 29694256

[71] Akiyama M Yasuoka H Yamaoka K Suzuki K Kaneko Y Kondo H. Enhanced IgG4 production by follicular helper 2 T cells and the involvement of follicular helper 1 T cells in the pathogenesis of IgG4-related disease. Arthritis Res Ther. (2016) 18:167. doi: 10.1186/s13075-016-1064-4 27411315 PMC4944254

[72] Wollenberg I Agua-Doce A Hernández A Almeida C Oliveira VG Faro J. Regulation of the germinal center reaction by Foxp3+ follicular regulatory T cells. J Immunol. (2011) 187:4553–60. doi: 10.4049/jimmunol.1101328 21984700

[73] Laidlaw BJ Lu Y Amezquita RA Weinstein JS Vander Heiden JA Gupta NT. Interleukin-10 from CD4(+) follicular regulatory T cells promotes the germinal center response. Sci Immunol. (2017) 2(16):eaan4767. doi: 10.1126/sciimmunol.aan4767 29054998 PMC5846620

[74] Ito F Kamekura R Yamamoto M Takano K Takaki H Yabe H. IL-10(+) T follicular regulatory cells are associated with the pathogenesis of IgG4-related disease. Immunol Lett. (2019) 207:56–63. doi: 10.1016/j.imlet.2019.01.008 30658078

[75] Rao DA Gurish MF Marshall JL Slowikowski K Fonseka CY Liu Y. Pathologically expanded peripheral T helper cell subset drives B cells in rheumatoid arthritis. Nature. (2017) 542:110–4. doi: 10.1038/nature20810 PMC5349321 28150777

[76] Pitzalis C Jones GW Bombardieri M Jones SA. Ectopic lymphoid-like structures in infection, cancer and autoimmunity. Nat Rev Immunol. (2014) 14:447–62. doi: 10.1038/nri3700 24948366

[77] Yabe H Kamekura R Yamamoto M Murayama K Kamiya S Ikegami I. Cytotoxic Tph-like cells are involved in persistent tissue damage in IgG4-related disease. Mod Rheumatol. (2021) 31:249–60. doi: 10.1080/14397595.2020.1719576 32023137

[78] Aoyagi R Maehara T Koga R Munemura R Tomonaga T Murakami Y. Single-cell transcriptomics reveals granzyme K-expressing cytotoxic Tfh cells in tertiary lymphoid structures in IgG4-RD. J Allergy Clin Immunol. (2024) 153:513–520.e10. doi: 10.1016/j.jaci.2023.08.019 37652139

[79] Kusuda T Uchida K Miyoshi H Koyabu M Satoi S Takaoka M. Involvement of inducible costimulator- and interleukin 10-positive regulatory T cells in the development of IgG4-related autoimmune pancreatitis. Pancreas. (2011) 40:1120–30. doi: 10.1097/MPA.0b013e31821fc796 21926547

[80] Kawamura E Hisano S Nakashima H Takeshita M Saito T. Immunohistological analysis for immunological response and mechanism of interstitial fibrosis in IgG4-related kidney disease. Mod Rheumatol. (2015) 25:571–8. doi: 10.3109/14397595.2014.1001474 25547019

[81] Tsuboi H Miyake K Moriyama M Tanaka A Watanabe M Abe Y. Analysis of IgG4 class switch-related molecules in IgG4-related disease. Arthritis Res Ther. (2012) 14:R171. doi: 10.1186/ar3924 22824292 PMC3580565

[82] Nakashima H Mahajan VS Maehara T Deshpande V Della-Torre E Wallace ZS. An amplification of IL-10 and TGF-beta in patients with IgG4-related tubulointerstitial nephritis. Clin Nephrol. (2010) 73:385–91. doi: 10.5414/cnp73385 20420800

[83] Mattoo H Mahajan VS Maehara T Deshpande V Della-Torre E Wallace ZS. Clonal expansion of CD4(+) cytotoxic T lymphocytes in patients with IgG4-related disease. J Allergy Clin Immunol. (2016) 138:825–38. doi: 10.1016/j.jaci.2015.12.1330 PMC5014627 26971690

[84] Maehara T Mattoo H Ohta M Mahajan VS Moriyama M Yamauchi M. Lesional CD4+ IFN-γ+ cytotoxic T lymphocytes in IgG4-related dacryoadenitis and sialoadenitis. Ann Rheum Dis. (2017) 76:377–85. doi: 10.1136/annrheumdis-2016-209139 PMC5435236 27358392

[85] Suzuki K Tamaru J Okuyama A Kameda H Amano K Nagasawa H. IgG4-positive multi-organ lymphoproliferative syndrome manifesting as chronic symmetrical sclerosing dacryo-sialadenitis with subsequent secondary portal hypertension and remarkable IgG4-linked IL-4 elevation. Rheumatol (Oxford). (2010) 49:1789–91. doi: 10.1093/rheumatology/keq113 20444858

[86] Sasaki T Yajima T Shimaoka T Ogawa S Saito T Yamaoka K. Synergistic effect of IgG4 antibody and CTLs causes tissue inflammation in IgG4-related disease. Int Immunol. (2020) 32:163–74. doi: 10.1093/intimm/dxz073 31713611

[87] Cai S Chen Y Hu Z Zhou T Huang Y Lin S. The landscape of T and B lymphocytes interaction and synergistic effects of Th1 and Th2 type response in the involved tissue of IgG4-RD revealed by single cell transcriptome analysis. J Autoimmun. (2022) 133:102944. doi: 10.1016/j.jaut.2022.102944 36401985

[88] Ohta N Makihara S Okano M Kurakami K Ishida A Furukawa T. Roles of IL-17, Th1, and Tc1 cells in patients with IgG4-related sclerosing sialadenitis. Laryngoscope. (2012) 122:2169–74. doi: 10.1002/lary.23429 22786718

[89] Yang Y Wang C Shi L Yang S Liu Y Luo J. Clinical characteristics and CD4(+) T cell subsets in igG4-related disease. Front Immunol. (2022) 13:825386. doi: 10.3389/fimmu.2022.825386 35432312 PMC9010737

[90] Akitake R Watanabe T Zaima C Uza N Ida H Tada S. Possible involvement of T helper type 2 responses to Toll-like receptor ligands in IgG4-related sclerosing disease. Gut. (2010) 59:542–5. doi: 10.1136/gut.2009.200972 20332525

[91] Wu X Peng Y Li J Zhang P Liu Z Lu H. Single-cell sequencing of immune cell heterogeneity in igG4-related disease. Front Immunol. (2022) 13:904288. doi: 10.3389/fimmu.2022.904288 35693817 PMC9184520

[92] Khosroshahi A Wallace ZS Crowe JL Akamizu T Azumi A Carruthers MN. International consensus guidance statement on the management and treatment of igG4-related disease. Arthritis Rheumatol. (2015) 67:1688–99. doi: 10.1002/art.39132 25809420

[93] Della-Torre E Bozzalla-Cassione E Sciorati C Ruggiero E Lanzillotta M Bonfiglio S. A CD8α- subset of CD4+SLAMF7+ Cytotoxic T cells is expanded in patients with igG4-related disease and decreases following glucocorticoid treatment. Arthritis Rheumatol. (2018) 70:1133–43. doi: 10.1002/art.40469 PMC6019645 29499100

[94] Masamune A Nishimori I Kikuta K Tsuji I Mizuno N Iiyama T. Randomised controlled trial of long-term maintenance corticosteroid therapy in patients with autoimmune pancreatitis. Gut. (2017) 66:487–94. doi: 10.1136/gutjnl-2016-312049 27543430

[95] Wu Q Chang J Chen H Chen Y Yang H Fei Y. Efficacy between high and medium doses of glucocorticoid therapy in remission induction of IgG4-related diseases: a preliminary randomized controlled trial. Int J Rheum Dis. (2017) 20:639–46. doi: 10.1111/1756-185X.13088 28556584

[96] Yamamoto M Yajima H Takahashi H Yokoyama Y Ishigami K Shimizu Y. Everyday clinical practice in IgG4-related dacryoadenitis and/or sialadenitis: results from the SMART database. Mod Rheumatol. (2015) 25:199–204. doi: 10.3109/14397595.2014.950036 25159154

[97] Lanzillotta M Campochiaro C Mancuso G Ramirez GA Capurso G Falconi M. Clinical phenotypes of IgG4-related disease reflect different prognostic outcomes. Rheumatol (Oxford). (2020) 59:2435–42. doi: 10.1093/rheumatology/keaa221 32591828

[98] Luo X Peng Y Zhang P Li J Liu Z Lu He. Comparison of the effects of cyclophosphamide and mycophenolate mofetil treatment against immunoglobulin G4-related disease: A retrospective cohort study. Front Med (Lausanne). (2020) 7:253. doi: 10.3389/fmed.2020.00253 32733900 PMC7358520

[99] Yunyun F Yu C Panpan Z Hua C Di W Lidan Z. Efficacy of Cyclophosphamide treatment for immunoglobulin G4-related disease with addition of glucocorticoids. Sci Rep. (2017) 7:6195. doi: 10.1038/s41598-017-06520-5 28733656 PMC5522435

[100] Lanzillotta M Della-Torre E Wallace ZS Stone JH Karadag O Fernández- Codina A. Efficacy and safety of rituximab for IgG4-related pancreato-biliary disease: A systematic review and meta-analysis. Pancreatology. (2021) 21:1395–401. doi: 10.1016/j.pan.2021.06.009 34244040

[101] Carruthers MN Topazian MD Khosroshahi A Witzig TE Wallace ZS Hart PA. Rituximab for IgG4-related disease: a prospective, open-label trial. Ann Rheum Dis. (2015) 74:1171–7. doi: 10.1136/annrheumdis-2014-206605 25667206

[102] Kaegi C Wuest B Schreiner J Steiner UC Vultaggio A Matucci A. Systematic review of safety and efficacy of rituximab in treating immune-mediated disorders. Front Immunol. (2019) 10:1990. doi: 10.3389/fimmu.2019.01990 31555262 PMC6743223

[103] Mancuso G Jofra T Lanzillotta M Aiuti A Cicalese MP di Colo G. Persistence of circulating T-follicular helper cells after rituximab is associated with relapse of IgG4-related disease. Rheumatol (Oxford). (2021) 60:3947–9. doi: 10.1093/rheumatology/keab344 33856429

[104] Akiyama M Kaneko Y. Comment on: Persistence of circulating T-follicular helper cells after rituximab is associated with relapse of IgG4-related disease. Rheumatology. (2021) 60:e412–3. doi: 10.1093/rheumatology/keab687 34505901

[105] Aletaha D Smolen JS. Diagnosis and management of rheumatoid arthritis: A review. Jama. (2018) 320:1360–72. doi: 10.1001/jama.2018.13103 30285183

[106] Pan L Lu MP Wang JH Xu M Yang SR. Immunological pathogenesis and treatment of systemic lupus erythematosus. World J Pediatr. (2020) 16:19–30. doi: 10.1007/s12519-019-00229-3 30796732 PMC7040062

[107] Matza MA Perugino CA Harvey L Fernandes AD Wallace ZS Liu H. Abatacept in IgG4-related disease: a prospective, open-label, single-arm, single-centre, proof-of-concept study. Lancet Rheumatol. (2022) 4:e105–12. doi: 10.1016/S2665-9913(21)00359-3 PMC9004478 35425928

[108] Mavragani CP Moutsopoulos HM. Sjögren's syndrome: Old and new therapeutic targets. J Autoimmun. (2020) 110:102364. doi: 10.1016/j.jaut.2019.102364 31831255

[109] Della-Torre E Lanzillotta M Yacoub MR. Dupilumab as a potential steroid-sparing treatment for IgG4-related disease. Ann Rheum Dis. (2022) 81:e24. doi: 10.1136/annrheumdis-2020-216945 31937521

[110] Matera MG Rogliani P Calzetta L Cazzola M. TSLP inhibitors for asthma: Current status and future prospects. Drugs. (2020) 80:449–58. doi: 10.1007/s40265-020-01273-4 32078149

[111] Perugino C Culver EL Khosroshahi A Zhang W Della-Torre E Okazaki K. Efficacy and safety of inebilizumab in igG4-related disease: Protocol for a randomized controlled trial. Rheumatol Ther. (2023) 10(6):1795–808. doi: 10.1007/s40744-023-00593-7 PMC10654302 37792260

[112] Katayama Y Katsuyama T Shidahara K Nawachi S Asano Y Ohashi K. A case of recurrent IgG4-related disease successfully treated with belimumab after remission of systemic lupus erythematosus. Rheumatol (Oxford). (2022) 61:e308–10. doi: 10.1093/rheumatology/keac284 35595251

[113] Kolev M Sarbu AC Möller B Maurer B Kollert F Semmo N. Belimumab treatment in autoimmune hepatitis and primary biliary cholangitis - a case series. J Transl Autoimmun. (2023) 6:100189. doi: 10.1016/j.jtauto.2023.100189 36718275 PMC9883290

[114] Stone JH Wallace Z Perugino CA Fernandes AD Patel P Foster PA. Final results of an open label phase 2 study of a reversible B cell inhibitor, Xmab5871, in IgG4-related disease. Arthritis Rheumatol. (2017) 69(Suppl. 10). doi: 10.1136/annrheumdis-2017-eular.3301

[115] Peng Q Wu F Shi Y Wang J Zhai Z Wang Z. Idiopathic multicentric castleman's disease mimicking immunoglobulin G4-related disease responding well to Bortezomib: a case report. BMC Nephrol. (2023) 24:290. doi: 10.1186/s12882-023-03335-7 37784011 PMC10546740

[116] Comdühr S Dübbers A Tharun L Graßhoff H Stone J Pitann S. Immunological changes and prevention of disease progression through elotuzumab therapy in refractory IgG4-related sclerosing mesenteritis. Rheumatol (Oxford). (2022) 61:e334–6. doi: 10.1093/rheumatology/keac302 35595249

[117] Mamizu H Ohta T Yanai K Yamazaki R Mamizu M Ishikawa D. Refractory eosinophilic granulomatosis with polyangiitis complicated with igG4-related disease showing different treatment responses for each organ. Intern Med. (2023) 62:2995–3000. doi: 10.2169/internalmedicine.1302-22 36823081 PMC10641191

[118] Jansen DT el Bannoudi H Arens R Habets KL Hameetman M Huizinga TW. Abatacept decreases disease activity in a absence of CD4(+) T cells in a collagen-induced arthritis model. Arthritis Res Ther. (2015) 17:220. doi: 10.1186/s13075-015-0731-1 26290328 PMC4545927

[119] Yamamoto M Takahashi H Takano K Shimizu Y Sakurai N Suzuki C. Efficacy of abatacept for IgG4-related disease over 8 months. Ann Rheum Dis. (2016) 75:1576–8. doi: 10.1136/annrheumdis-2016-209368 27147710

[120] Nakayamada S Kubo S Yoshikawa M Miyazaki Y Yunoue N Iwata S. Differential effects of biological DMARDs on peripheral immune cell phenotypes in patients with rheumatoid arthritis. Rheumatol (Oxford). (2018) 57:164–74. doi: 10.1093/rheumatology/kex012 28371836

[121] Carvajal Alegria G Pochard P Pers JO Cornec D. Could abatacept directly target expanded plasmablasts in IgG4-related disease? Ann Rheum Dis. (2016) 75:e73. doi: 10.1136/annrheumdis-2016-210400 27624790

[122] Cheng LE Amoura Z Cheah B Hiepe F Sullivan BA Zhou L. Brief report: A randomized, double-blind, parallel-group, placebo-controlled, multiple-dose study to evaluate AMG 557 in patients with systemic lupus erythematosus and active lupus arthritis. Arthritis Rheumatol. (2018) 70:1071–6. doi: 10.1002/art.40479 PMC6032945 29513931

[123] Sullivan BA Tsuji W Kivitz A Peng J Arnold GE Boedigheimer MJ. Inducible T-cell co-stimulator ligand (ICOSL) blockade leads to selective inhibition of anti-KLH IgG responses in subjects with systemic lupus erythematosus. Lupus Sci Med. (2016) 3:e000146. doi: 10.1136/lupus-2016-000146 27099766 PMC4836284

[124] Zhang M Lee F Knize A Jacobsen F Yu S Ishida K. Development of an ICOSL and BAFF bispecific inhibitor AMG 570 for systemic lupus erythematosus treatment. Clin Exp Rheumatol. (2019) 37:906–14. 30789152

[125] Abuqayyas L Chen PW Dos Santos MT Parnes JR Doshi S Dutta S. Pharmacokinetics and pharmacokinetic/pharmacodynamic properties of rozibafusp alfa, a bispecific inhibitor of BAFF and ICOSL: analyses of phase I clinical trials. Clin Pharmacol Ther. (2023) 114:371–80. doi: 10.1002/cpt.2929 37150935

[126] Fu Y Lin Q Zhang Z Zhang L. Therapeutic strategies for the costimulatory molecule OX40 in T-cell-mediated immunity. Acta Pharm Sin B. (2020) 10:414–33. doi: 10.1016/j.apsb.2019.08.010 PMC7049610 32140389

[127] Harb H Chatila TA. Mechanisms of dupilumab. Clin Exp Allergy. (2020) 50:5–14. doi: 10.1111/cea.13491 31505066 PMC6930967

[128] Pelaia C Vatrella A Gallelli L Terracciano R Navalesi P Maselli R. Dupilumab for the treatment of asthma. Expert Opin Biol Ther. (2017) 17:1565–72. doi: 10.1080/14712598.2017.1387245 28990423

[129] Lipworth B Chan R Kuo CR. Dupilumab for nasal polyposis. Lancet. (2020) 396:233. doi: 10.1016/S0140-6736(20)30562-6 32711789

[130] Beck LA Thaçi D Hamilton JD Graham NM Bieber T Rocklin R. Dupilumab treatment in adults with moderate-to-severe atopic dermatitis. N Engl J Med. (2014) 371:130–9. doi: 10.1056/NEJMoa1314768 25006719

[131] Kanda M Kamekura R Sugawara M Nagahata K Suzuki C Takano K. IgG4-related disease administered dupilumab: case series and review of the literature. RMD Open. (2023) 9(1):e003026. doi: 10.1136/rmdopen-2023-003026 36894196 PMC10008221

[132] Simpson RS Lau SKC Lee JK. Dupilumab as a novel steroid-sparing treatment for IgG4-related disease. Ann Rheum Dis. (2020) 79:549–50. doi: 10.1136/annrheumdis-2019-216368 31857343

[133] Ebbo M De Sainte-Marie B Muller R Piperoglou C Grados A Vély F. Correspondence on: 'Dupilumab as a novel steroid-sparing treatment for IgG(4)-related disease' by Simpson et al. Ann Rheum Dis. (2022) 81:e26. doi: 10.1136/annrheumdis-2020-217010 31996366

[134] Corren J Ziegler SF. TSLP: from allergy to cancer. Nat Immunol. (2019) 20:1603–9. doi: 10.1038/s41590-019-0524-9 31745338

[135] Hillen MR Radstake TR Hack CE van Roon JA. Thymic stromal lymphopoietin as a novel mediator amplifying immunopathology in rheumatic disease. Rheumatol (Oxford). (2015) 54:1771–9. doi: 10.1093/rheumatology/kev241 26163286

[136] Yajima R Takano K Konno T Kohno T Kaneko Y Kakuki T. Mechanism of fibrogenesis in submandibular glands in patients with IgG4-RD. J Mol Histol. (2018) 49:577–87. doi: 10.1007/s10735-018-9796-x 30251185

[137] Lu H Wu X Peng Y Sun R Nie Y Li J. TSLP promoting B cell proliferation and polarizing follicular helper T cell as a therapeutic target in IgG4-related disease. J Transl Med. (2022) 20:414. doi: 10.1186/s12967-022-03606-1 36076269 PMC9461269

[138] Kurihara M Kabata H Irie M Fukunaga K. Current summary of clinical studies on anti-TSLP antibody, Tezepelumab, in asthma. Allergol Int. (2023) 72:24–30. doi: 10.1016/j.alit.2022.11.006 36470789

[139] Seleznik GM Reding T Romrig F Saito Y Mildner A Segerer S. Lymphotoxin β receptor signaling promotes development of autoimmune pancreatitis. Gastroenterology. (2012) 143:1361–74. doi: 10.1053/j.gastro.2012.07.112 22863765

[140] Pan Z Zhu T Liu Y Zhang N. Role of the CXCL13/CXCR5 axis in autoimmune diseases. Front Immunol. (2022) 13:850998. doi: 10.3389/fimmu.2022.850998 35309354 PMC8931035

[141] Jamilloux Y El Jammal T Vuitton L Gerfaud-Valentin M Kerever S Sève P. JAK inhibitors for the treatment of autoimmune and inflammatory diseases. Autoimmun Rev. (2019) 18:102390. doi: 10.1016/j.autrev.2019.102390 31520803

[142] Khan S Gordins P Durairaj S. JAK inhibition as a therapeutic strategy for igG4-RD. J Investig Allergol Clin Immunol. (2021) 31:280–1. doi: 10.18176/jiaci 33237022

[143] Khanna D Padilla C Tsoi LC Nagaraja V Khanna PP Tabib T. Tofacitinib blocks IFN-regulated biomarker genes in skin fibroblasts and keratinocytes in a systemic sclerosis trial. JCI Insight. (2022) 7(17):e159566. doi: 10.1172/jci.insight.159566 35943798 PMC9536259

[144] Xia CS Long Y Liu Y Alifu A Zeng X Liu C. IL-7 promotes the expansion of circulating CD28- cytotoxic T lymphocytes in patients with igG4-related disease via the JAK signaling. Front Immunol. (2022) 13:922307. doi: 10.3389/fimmu.2022.922307 35874706 PMC9301466

[145] Bai Y Wang W Yin P Gao J Na L Sun Y. Ruxolitinib alleviates renal interstitial fibrosis in UUO mice. Int J Biol Sci. (2020) 16:194–203. doi: 10.7150/ijbs.39024 31929748 PMC6949153

[146] You H Xu D Zhao J Li J Wang Q Tian X. JAK inhibitors: prospects in connective tissue diseases. Clin Rev Allergy Immunol. (2020) 59:334–51. doi: 10.1007/s12016-020-08786-6 32222877

